# Therapeutic Effects of Melatonin Receptor Agonists on Sleep and Comorbid Disorders

**DOI:** 10.3390/ijms150915924

**Published:** 2014-09-09

**Authors:** Moshe Laudon, Anat Frydman-Marom

**Affiliations:** Neurim Pharmaceuticals Ltd., 27 Habarzel St. Tel-Aviv 6971039, Israel; E-Mail: anatf@neurim.com

**Keywords:** insomnia comorbid, sleep, melatonin receptors agonists

## Abstract

Several melatonin receptors agonists (ramelteon, prolonged-release melatonin, agomelatine and tasimelteon) have recently become available for the treatment of insomnia, depression and circadian rhythms sleep-wake disorders. The efficacy and safety profiles of these compounds in the treatment of the indicated disorders are reviewed. Accumulating evidence indicates that sleep-wake disorders and co-existing medical conditions are mutually exacerbating. This understanding has now been incorporated into the new Diagnostic and Statistical Manual of Mental Disorders, 5th Edition (DSM-5). Therefore, when evaluating the risk/benefit ratio of sleep drugs, it is pertinent to also evaluate their effects on wake and comorbid condition. Beneficial effects of melatonin receptor agonists on comorbid neurological, psychiatric, cardiovascular and metabolic symptomatology beyond sleep regulation are also described. The review underlines the beneficial value of enhancing physiological sleep in comorbid conditions.

## 1. Introduction

Insomnia is defined as difficulty in initiating and/or maintaining sleep and/or non-restorative sleep, associated with some type of daytime impairment or distress. The Diagnostic and Statistical Manual of Mental Disorders, 4th Edition (DSM-4) considered the sleep disorder as “primary insomnia” if it occurs as an independent disorder and “secondary insomnia” when it relates to another mental disorder (e.g., depression) or medical condition (e.g., pain). The new Diagnostic and Statistical Manual of Mental Disorders, 5th Edition (DSM-5) pays more attention to co-existing medical conditions when it comes to sleep disorders (now called sleep-wake disorders), to better emphasize when an individual has a sleep disorder warranting independent clinical attention, in addition to any medical and mental disorders that are also present (http://pro.psychcentral.com/dsm-5-changes-sleep-wake-disorders/004414.html). DSM-5 recognizes that co-existing medical conditions, mental disorders and sleep disorders (comorbid insomnia) are interactive and bidirectional and it is not as important to make assumptions about what causes the sleep disorder [1]. That is the reason the diagnosis of primary insomnia has been renamed insomnia disorder, in order to avoid the differentiation of primary and secondary insomnia. Insomnia can be transient, lasting a few days or weeks. Or, if it lasts three months or more, it can be a chronic disorder, and in such cases it will typically not remit spontaneously. Insomnia is a highly prevalent sleep disorder throughout the world, approximately 10% of the world’s population will report that they suffer from chronic or persistent insomnia [2]. Various studies suggest that the vast majority of insomnia patients seen in psychiatric practices, and about 50% of those seen in primary care practices, have comorbid conditions [3].

Previous drug developments for insomnia focused on primary insomnia and clinical trials typically recruited patients with the primary disorder. However, in the majority of cases insomnia is comorbid with other conditions and the presence of such comorbidities was not given due attention in the development of insomnia drugs. It becomes increasingly recognized in recent years that insomnia increases the risk of hypertension, cardiovascular disease, metabolic disorders and Alzheimer’s dementia and the presence of these medical conditions exacerbate the insomnia [2,4]. The change in the definition of insomnia by DSM-5 will most likely change the approach to insomnia drug development.

Benzodiazepine receptor agonists e.g., zolpidem, zaleplon, zopiclone, and eszopiclone (z-drugs) are the most commonly prescribed medication class for managing insomnia. Benzodiazepines and related nonbenzodiazepine drugs with effects similar to benzodiazepines (z-drugs) have shown evidence of impairing cognitive and psychomotor skills, and increasing risk of falls, rebound, dependence or abuse potential and significant adverse events compared to the placebo. This has raised concern about the association of their use with considerable morbidity and mortality. For these reasons, the use of benzodiazepine receptor agonists is in decline and they are not recommended for use in people with Alzheimer’s disease and other comorbid disorders [5,6].

Of specific relevance is the question whether the insomnia drug has effects on comorbidities associated with insomnia, including altered mood, depression (psychiatric symptoms), increased sensitivity to pain, memory impairment and headaches (neurological symptoms), hypertension, myocardial diseases (cardiovascular symptoms), diabetes, metabolic syndrome and dyslipidemia (metabolic symptoms).

Melatonin (*N*-acetyl-5-methoxytryptamine), the hormone normally secreted from the pineal gland at night, serves as the signal of darkness in the organism and as such plays a pivotal role in the physiological regulation of circadian rhythms, including sleep [7]. The circadian rhythm of synthesis and secretion of melatonin is closely associated with the sleep rhythm in both sighted and blind subjects [8]. Daytime administration of exogenous melatonin (when it is not present endogenously) promotes sleep in humans and results in sleep-like brain activity patterns at specific areas such as the precuneus and hippocampus [9]. Endogenous melatonin levels decrease with age [10] and this decline may contribute to the common complaint of poor sleep quality in elderly people [11]. Sleep disorders in children with autism spectrum disorder and some neurogenetic disorders have also been linked to insufficient or abnormal melatonin rhythms [12,13,14,15]. Other sleep disorders, such as delayed sleep disorders or non-24 h sleep-wake disorders in blind people are attributed to problems in synchronization of the endogenous circadian clock with the environmental light-dark cycle and leading to shifted or unstable timing of melatonin secretion [16].

A number of melatonin receptors agonist have recently become available for treatment of sleep disorders: ramelteon for the treatment of insomnia characterized by difficulty with sleep onset, prolonged-release melatonin for treatment of primary insomnia characterized by poor quality of sleep in patients who are aged 55 or over, agomelatine for the treatment of depression and associated sleep disorder, and tasimelteon for the treatment of non-24 h sleep-wake disorder in the blind. This review summarizes the current evidence regarding the efficacy and safety of melatonin receptor agonists in the indicated sleep disorders and their potential effects on comorbid conditions.

## 2. Melatonin-Receptor-Agonists

### 2.1. Ramelteon

Ramelteon is a selective Melatonin receptor type 1 (MT1) and MT2 receptors agonist [17]. Ramelteon is absorbed rapidly (0.5–1.5 h) after oral administration, with 1.8% oral bioavailability due to prominent first pass effect (84% total absorption). It is metabolized in the liver and excreted mainly as glucuronide conjugates via urine and partly (up to 4%) via feces. Ramelteon has a half-life of 1.0 to 2.6 h, while the half-life of the active metabolite M-II extends to 2 to 5 h. The main ramelteon metabolite M-II has a weak MT1 and MT2 agonistic activity and a low serotonin 5-HT_2B_ receptor affinity; however, due to a relatively longer half-life it circulates at 10- to 100-fold higher concentrations compared to its parent substance which makes its effects of potential clinical relevance.

Ramelteon, in 8 mg tablets (Rozerem; Takeda Pharmaceuticals, North American, Deerfield, IL, USA) has been approved (2005) by the Food and Drug Administration )FDA (for the treatment of insomnia characterized by difficulty with sleep onset and is currently commercialized in the USA and Japan [18] (Summary of product characteristics, FDA approved label 10.20.08 NDA 21-782, S-008, S-009, S-010 http://www.accessdata.fda.gov/drugsatfda_docs/label/2008/021782s008s009s010lbl.pdf). In adults and older patients with chronic insomnia, ramelteon reduced latency to persistent sleep by 40 min (56%) from baseline at 6 months compared with 30 min (43%) in patients receiving placebo [19]. The incidence of adverse effects with ramelteon was comparable to the placebo, being free of next-day residual effects on psychomotor performance, memory, mood and feelings, or alertness and concentration. Because it does not cause dependence and addiction ramelteon is currently the only insomnia drug that is not classified as a scheduled hypnotic in the USA [20].

Ramelteon has recently been shown to be effective in the prevention of delirium in three recent controlled studies [21,22]. Additional studies to confirm the therapeutic benefits of ramelteon are warranted.

### 2.2. Circadin

Prolonged-release melatonin (Circadin; Neurim Pharmaceuticals, Tel Aviv, Israel) is the first melatonin receptor agonist licensed in the European Union (2007) indicated for the treatment of primary insomnia in patients aged 55 years and older. There is an age-related decline in the robustness of the biological clock and melatonin production, thus depriving the brain of an important sleep regulator [23,24,25]. In patients aged 55 years and over who suffer from poor sleep quality; melatonin production is even lower than in healthy elderly people without such complaints [11,24].

Circadin is designed to mimic the release pattern of endogenous melatonin by releasing melatonin gradually over 8–10 h [26,27]. It is fully absorbed but bioavailability reaches 15% due to high first-pass hepatic degradation primarily through CYP1A2. Following oral ingestion maximal melatonin concentrations in the blood are attained after 0.75 h in the fasting and 3 h in fed condition, after meal. Circadin is eliminated mainly as sulfated and glucuronide conjugates after biotransformation in the liver and only partly (2%) as unchanged melatonin. The terminal half-life of Circadin is 3.5–4.0 h (Summary of product characteristics, Circadin, Approved EMA, June 2007 http://www.ema.europa.eu/docs/en_GB/document_library/EPAR_Summary_for_the_public/human/000695/WC500026805.pdf).

Circadin was approved in Europe by the European medicines agency (EMA) 2007 as monotherapy for the short-term treatment of primary insomnia characterized by poor quality of sleep in patients who are aged 55 or over and is currently commercialized in Europe and Asia-Pacific territories. It has been shown to significantly improve sleep latency, quality of sleep, quality of life and morning alertness in primary insomnia patients, suggesting more restorative sleep, without withdrawal symptoms upon discontinuation [26,27,28,29,30].

The safety and tolerability profile of Circadin in clinical trials was comparable to placebo group, with no negative effects on memory or postural stability during the night. Based on long term efficacy and safety data, treatment with Circadin was not limited to 2–4 weeks as with classical sedative hypnotics sleep drugs but allowed for up to 3 months without interruption [26,27].

### 2.3. Agomelatine

Agomelatine (Valdoxan, Melitor, Thymanax, Servier Pharmaceuticals, Neuilly-sur-Seine, France) is a melatonin MT1 and MT2 receptors agonist and a weak 5-HT_2C_ antagonist.

After ingestion, agomelatine is rapidly (80%) absorbed irrespective of food intake. The peak plasma levels are reached in 1–2 h. It is highly protein bound (>95%) and is metabolized in the liver, primarily through CYP1A2, to form inactive metabolites that are mainly (80%) excreted in the urine. (Summary of product characteristics, Thymanax, Approved EMA, February 2009. http://www.ema.europa.eu/ema/index.jsp?curl=pages/medicines/human/medicines/000916/human_med_001093.jsp&mid=WC0b01ac058001d124).

Agomelatine received marketing authorization in the European Union in 2009, for the treatment of depression. In patients with major depression, agomelatine was similarly as effective as paroxetine, sertraline, venlafaxine and fluoxetine, with a lower relapse rate (23.9%) compared to the placebo (50.0%) [31,32].

A recent meta-analysis that included unpublished studies reported a greater reduction in symptoms scores with agomelatine than with the placebo and some other antidepressants, but the size of the benefit looks small and may not be clinically relevant [33]. Recent recommendations advise that agomelatine is appropriate as an alternative second line agent in the pharmacological treatment of severe major depression [34].

In addition, agomelatine improved sleep quality and reduced waking after sleep onset in depressive patients [35,36]. While the sleep promoting effects of agomelatine can clearly be ascribed to its melatoninergic activity, the antidepressant function has been ascribed to 5-HT_2C_ receptor antagonism. However, due to agomelatine’s short half-life and low 5-HT_2C_ affinity, it is disputed to what extent the 5-HT_2C_ antagonism contributes to its therapeutic effect [37]. At therapeutic doses, it preserved vigilance and memory in healthy volunteers, with no sedation in the morning following drug intake and demonstrates lower rates of sexual dysfunction and discontinuation compared to some other antidepressants [38]. Due to the risk of common liver enzymes elevation and rare serious liver reactions, routine laboratory monitoring of liver function is recommended periodically throughout treatment [39].

### 2.4. Tasimelteon

Tasimelteon (Hetlioz; Vanda Pharmaceuticals, Washington, DC, USA) is a specific MT1 and MT2 receptors agonist. The peak concentration (Tmax) of tasimelteon occurred approximately 0.5 to 3 h after fasted oral administration. Metabolism of tasimelteon consists primarily of oxidation at multiple sites and oxidative dealkylation. CYP1A2 and CYP3A4 are the major isozymes involved in the metabolism of tasimelteon. Phenolic glucuronidation is the major phase II metabolic route. Major metabolites had 13-fold or less activity at melatonin receptors compared to tasimelteon.

Tasimelteon has been developed for the treatment of circadian rhythms sleep disorders [40,41] and approved in the USA in 2014 for the treatment of non-24 h sleep-wake disorder in the blind [42]. Approximately 55%–70% of totally blind patients are thought to have non-entrained rhythms and if they report periodic difficulties with sleep or daytime alertness they are diagnosed as having non-24 h (“free running”) sleep-wake disorder (N24HSWD).

Phase II and III studies have been conducted in transient insomnia associated with shifted sleep and wake time [41,43]. In the phase II study, tasimelteon reduced sleep latency; increased sleep efficiency compared with placebo and dose-dependently advanced plasma melatonin rhythm. In the phase III study, tasimelteon improved sleep latency, sleep efficiency and wake after sleep onset. The frequency of adverse events in both studies was comparable to placebo [44].

## 3. Preclinical and Clinical Evidence on Effects of Melatonin Receptor Agonists on Comorbid Conditions

### 3.1. Comorbid Psychiatric Diseases

A bidirectional link exists between mental illness and sleep disorders. Disturbances of the circadian clock can result in neurobiological dysfunction which in turn can be manifest as depressive symptoms; on the other hand, mood decline can affect the circadian system and cause insomnia and circadian rhythm sleep disorders [45,46]. The sleep disorder can exacerbate the mental disease. Evidences in the literature have demonstrated that 60%–80% of patients with major depression suffer from sleep changes, especially insomnia [47,48,49]. The odds of having at least one psychiatric diagnosis is 5.04 to 6 times greater in patients with severe insomnia as compared to those without insomnia and increasing insomnia severity is associated with increased chronic medical and psychiatric illnesses [49,50]. Insomnia has been confirmed as a risk factor for future mental illness [51] where individuals who report insomnia or poor quality of sleep may be at higher risk for relapse of depression throughout their lifetime [52,53]. Furthermore, patients with psychiatric diagnoses (including depression, post-traumatic stress disorder (PTSD), schizophrenia and comorbid sleep disturbances were significantly more likely to report suicidal behavior [54].

Many antidepressants have untoward adverse effects on sleep, particularly causing or worsening insomnia, daytime sleepiness or sedation. Given the bidirectional relationship between psychiatric illness and insomnia, a medication that improves sleep quality may be a rational approach for improving the medical condition of psychiatric patients and in particular improve the quality of life of depresses subjects [55,56].

### 3.2. Melatonin Receptor Agonists in Comorbid Psychiatric Diseases: Preclinical Evidence

Melatonin receptor agonists have shown to exert antidepressant and/or anxiolytic effects in animal models. The exact mechanisms underlying the antidepressant and anxiolytic activities of melatonin agonists are not well established but γ-aminobutiric acid (GABA), serotonin (5-HT), *N*-methyl-d-aspartate (NMDA) receptors and the l-arginine/NO pathways have been implicated in mediating some brain melatonin effects [57,58,59,60,61]. Interestingly, melatonin administration has been shown to enhance GABA-benzodiazepine binding to brain membranes [62,63] and increase of GABA turnover rate and GABA-induced chloride influx [64,65].

Agomelatine, a potent melatonin receptor agonist, also acts as an antagonist at 5-HT_2C_ receptors and exerts antidepressant activities in several animal models [66,67,68]. This action has been implicated in its antidepressant activity because several antidepressants such as mianserin, mirtazapine and trazodone display moderate to high affinity for 5-HT_2C_ receptors [69]. Melatonin has been shown to act as a 5-HT_2A_ antagonist [70] and regulate both spontaneous efflux and evoked release of serotonin in the rat hippocampus [71]. These studies suggest that an interaction with the central serotonergic system is involved in the antidepressant and anxiolytic activities of melatonin and its analogues [72].

### 3.3. Melatonin Receptor Agonists in Comorbid Psychiatric Diseases: Clinical Evidence

#### 3.3.1. Melatonin

In schizophrenic patients with comorbid insomnia, melatonin (3 mg immediate release, nightly for 15 days, *n* = 40) significantly reduced sleep-onset latency, improved the quality and depth of nighttime sleep, reduced the number of nighttime awakenings and increased the duration of sleep without producing a morning hangover compared to placebo. Melatonin also significantly heightened freshness on awakening and improved mood and daytime functioning [73]. Circadin treatment (2 mg nightly for 7 weeks, *n* = 19, crossover) of schizophrenia patients with insomnia comorbidity significantly decreased sleep latency, improved sleep efficiency and increased sleep duration [74].

In patients with major depressive disorder add-on of slow-release melatonin (2.5–10 mg) to standard antidepressant treatment with fluoxetine demonstrated improvement in sleep compared to placebo but had no effect on the rate of improvement in depression symptoms [75,76].

#### 3.3.2. Agomelatine

The effectiveness of agomelatine in reducing sleep complaints of depressed patients has been evaluated. In an open label study, agomelatine (25 mg/day for 6 weeks) contributed to restore sleep architecture in depressed patients as shown by polysomnography records, improved sleep quality and continuity and increased slow-wave sleep (SWS) duration without modifying rapid eye movement (REM) sleep time [77].

In a comparator trial against venlafaxine (75–150 mg/day), agomelatine (25–50 mg/day) (6 weeks, *n* = 322) had comparable antidepressant efficacy but earlier and greater efficacy in improving subjective sleep than venlafaxine in depressed patients [78]. In addition, agomelatine reduced circadian rest-activity/sleep-wake cycle disturbances in depressed patients suggesting improvement in sleep and daytime functioning [79].

#### 3.3.3. Ramelteon

In anxiety disorder patients, ramelteon (8 mg, 12-week open-label, *n* = 27) showed significant improvement of sleep parameters (shorter latency, increased total sleep time (TST) and reduction of daytime sleepiness) as well as a reduction in anxiety symptoms [80].

In patients diagnosed with bipolar I disorder exhibiting manic symptoms and insomnia (ramelteon 8 mg/day, 8 weeks, double-blind, *n* = 21) there were no significant differences between ramelteon, added on as adjunctive treatment and placebo, in reducing symptoms of insomnia, mania, and global severity of illness. However, ramelteon, but not the placebo, was associated with improvement in a global rating of depressive symptoms [81].

In patients suffering from euthymic bipolar disorder and sleep disturbances, ramelteon, added on as adjunctive treatment (8 mg/day, 23 weeks double-blind, *n* = 83), was effective in maintaining stability in individuals with bipolar disorder. Patients treated with ramelteon were approximately half as likely to relapse as patients treated with placebo [82].

#### 3.3.4. Tasimelteon

In major depressive disorder (MDD) Tasimelteon (20 mg/day, 8 weeks, double blind, *n* = 507) showed no change from baseline in the Hamilton Depression Scale (HAMD-17) as compared to placebo. (http://www.clinicaltrials.gov/ct2/show/study/NCT01428661?term=Major+Depressive+Disorder++and+tasimelteon&rank = 1).

## 4. Melatonin Receptor Agonists in Comorbid Neurological Diseases

Alzheimer disease (AD) is the leading cause of dementia in the elderly population. AD is characterized by progressive loss of cognitive function, memory dysfunction and neuronal death. The degenerative process often produces neurobehavioral symptoms including sleep disturbances mainly characterized by night time awakenings and sleep-wake disturbances. The amount and quality of sleep declines with aging and to a greater extent in AD.

Parkinson’s disease (PD), the second most common form of neurodegenerative diseases after AD, affecting 1%–2% of the elderly population. Among the clinical features of PD are motor impairments involving resting tremor, bradykinesia, postural instability and rigidity along with non-motoric symptoms such as autonomic, cognitive and psychiatric problems [83,84,85]. Several studies have demonstrated the high prevalence of sleep disturbances in PD, which in some cases was close to 90% which is correlated to the severity of the disease [86,87].

Neurodegenerative diseases involving the central nervous system (CNS), e.g., AD and PD may impair sleep either as a result of the brain lesion or because of illness-related discomfort (motor immobility, social and familial impairment, depression, drugs). Some neurological conditions characterized by movement disorders that start or persist during sleep hinder sleep onset and/or sleep continuity, causing a poor sleep complaint [86]. While cognitive and motor symptoms are used to define AD and PD, respectively, patients with both disorders exhibit sleep disturbances including insomnia, hypersomnia and excessive daytime napping. The molecular basis of perturbed sleep in AD and PD may involve damage to hypothalamic and brainstem nuclei that control sleep-wake cycles [87]. Compelling evidence indicates a causal link between poor sleep and increased AD risk and memory loss [88,89,90]. Poor sleep quality or short sleep, increase β amyloid burden in brain areas typically affected in AD [91,92]. AD is associated with sleep disturbances in at least 25% to 35% of affected individuals [93]. A recent study on sleep quality and preclinical AD demonstrated that Aβ deposition in the preclinical stage of AD is associated with worse sleep quality but not with changes in sleep quantity [92].

The earliest and more commonly reported manifestations of Parkinson patients are difficulty initiating and maintaining restorative sleep, by either a reduction of stages 3/4 of non-REM sleep or by a decrease in REM sleep [94,95]. Other abnormalities are fragmented sleep with an increased number of arousals and awakenings and Parkinson-specific motor phenomena such as nocturnal immobility, rest tremor, eye blinking and dyskinesia [96,97,98]. Additionally, PD patients have been reported to experience significant excessive daytime somnolence [99,100].

The use of melatonin receptors agonists for sleep in neurodegenerative disorders is therefore of great potential importance.

### 4.1. Melatonin Receptor Agonists in Comorbid Neurological Diseases: Preclinical Evidence

Alzheimer pathogenesis involves mitochondrial dysfunction, abnormal tau phosphorylation, oxidative stress and apoptosis. In preclinical studies, *in vitro* and *in vivo* melatonin reversed some of these alterations and protected mitochondrial membranes from obvious damage [101]. In addition to its well-established antioxidant effect, melatonin prevents Aβ-mediated toxicity by inhibiting Aβ generation aggregation and formation of amyloid fibril [102]. Furthermore, melatonin attenuates tau hyper phosphorylation induced by activation of protein kinases [102] or inhibition of protein phosphatases and may be involved in the physiological regulation of tau phosphorylation [102,103,104,105].

There is considerable evidence that melatonin is neuroprotective in diverse models of Parkinson’s disease. Melatonin given systemically prevented apomorphine-induced circling behavior in 6-OHDA-lesioned rats [106] and potentiated low dose l-3,4-dihydroxyphenylalanine (L-DOPA) effects in 1-methyl-4-phenyl-1,2,3,6-tetrahydropyridine (MPTP)-induced experimental Parkinsonism in mice [107]. Melatonin administration was also found to counteract MPTP-induced lipid peroxidation in the striatum, hippocampal and midbrain regions [108] and prevent neuronal cell death in the nigrostriatal pathway [109].

Melatonin efficiently protects neuronal cells from Aβ-mediated toxicity via antioxidant and anti-amyloid properties. Studies have also demonstrated that melatonin efficiently attenuates Alzheimer-like tau hyperphosphorylation. Additionally, melatonin also plays a role in protecting the cholinergic system and in anti-inflammation [110]. Decreased level of melatonin can also attribute to AD pathology [111]. Both animal and human studies suggest that sleep disruption may contribute to cognitive impairment and AD progression.

### 4.2. Melatonin Receptor Agonists in Comorbid Neurological Diseases: Clinical Evidence

#### 4.2.1. Alzheimer Disease

It has been shown that the severity of mental and sleep impairments in demented people correlate significantly with the decrease in pineal melatonin production and cerebrospinal fluid (CSF) melatonin level [112].

Melatonin levels both in CSF and in postmortem human pineal gland are already reduced in preclinical AD subjects, who are still cognitively intact and have only the earliest signs of AD neuropathology [113,114].

Several studies, reported on the effects of immediate and prolonged release melatonin on sleep and cognition in AD.

A study of melatonin (3 mg, 4 weeks double blind, *n* = 20) reported on a significant improvement in actigraphy recorded sleep time and decreased activity in the night in AD patients. Significant improvements in ADAS-cog (Alzheimer’s Disease Assessment Scale-cognitive subscale) and ADAS-non-cog scales were also reported [115]. However, in another double blind test, placebo controlled study of immediate and sustained release melatonin (5-mg slow-release melatonin, or 10-mg melatonin, 2 month treatment, *n* = 157) non-significant trends for increased nocturnal total sleep time and decreased wake after sleep onset were observed in the melatonin groups relative to placebo using actigraphic measurements [116]. Caregiver subjective ratings of sleep quality showed improvement with treatment of 2.5 mg sustained-release melatonin for 2 months.

In another study, the combination therapy of melatonin, (5 mg/day, 10 weeks double blind placebo controlled, *n* = 50) and bright-light treatment in advanced dementia patients increased day wake time and activity levels and strengthened the rest-activity rhythm [117].

A double blind, placebo controlled trial of prolonged release melatonin ((PRM) 2 mg/day, 24 weeks double blind, *n* = 73) in patients with mild-to-moderate AD receiving standard therapy (acetylcholinesterase inhibitors with or without memantine) patients had significantly less cognitive decline than placebo as measured by IADL (instrumental activities of daily living) and MMSE (mini-mental state examination) and better sleep efficiency. In the subgroup of patients with comorbid insomnia (Pittsburgh sleep quality index (PSQI) > 6) PRM treatment resulted in significant and clinically meaningful effects *vs.* placebo in median ADAS-cog and mean IADL, MMSE and sleep efficiency [118].

Negative results were also published with the use of melatonin. No significant effects of melatonin (8.5 mg/day, immediate release and 1.5 sustained release, *n* = 44, treatment consist of 10 consecutive nights), compared to placebo were seen on sleep and circadian rhythms in a randomized placebo controlled study in institutionalized patients with AD [119]. Another double blind placebo controlled, cross over study (*n* = 25, participants with DSM-4 diagnoses of dementia with sleep disturbance, slow release melatonin, 6 mg/day, 7 weeks) showed no effect on median total sleep time, number of awakenings or sleep efficiency on melatonin in compared to placebo [120].

Mild cognitive impairment (MCI) is an etiologically heterogeneous syndrome characterized by cognitive impairment preceding dementia. Approximately 12% of MCI patients convert to Alzheimer’s disease or other dementia disorders every year.

Beneficial effects of melatonin administration in MCI patients were also reported. In a double blind placebo controlled crossover study in MCI elderly individuals with self-reported sleep-wake disturbances, melatonin (6 mg, *n* = 6), enhanced the rest-activity rhythm and reduced sleep onset latency and the number of transitions from sleep to wakefulness. However, total sleep time and wake after sleep onset were not improved. Nevertheless, the ability to remember previously learned items improved along with a significant reduction in depressed moods [121].

In a retrospective study on 150 MCI patients receiving daily 3–24 mg of immediate-release melatonin preparations at bedtime for up to 5 years, patients treated with melatonin exhibited significantly better performance in Mini-Mental State Examination, the cognitive subscale of the Alzheimer’s disease Assessment Scale, emotional performance and daily sleep/wake cycle [122].

To the best of our knowledge, there have not been published studies that report efficacy of ramelteon, agomelatine and tasimelteon in treatment of comorbid insomnia in patients suffering from Alzheimer disease. However, based on results obtained with melatonin in AD and MCI patients, melatonin receptor agonists are of potential benefit in the treatment of preclinical and early AD through improving sleep without impairing memory consolidation, and thereby improving cognitive functioning.

#### 4.2.2. Parkinson’s Disease

Some investigators regard PD as being related to a melatonin-dopamine imbalance (a “melatonin hyperplasia” disorder) and have hypothesized that melatonin antagonists would be beneficial [123,124]. However, there are reports on reduced MT1 and MT2 receptor expression in the striatum and other brain regions such as the amygdala which may curtail potential response to melatonin [125]. There are also inconsistent findings with both enhanced and decreased melatonin secretion in PD patients [126,127].

Several studies suggest beneficial effects of melatonin on sleep in PD patients. In one study, melatonin (5–50 mg/day, 2 weeks double blind, *n* = 40) significantly increased actigraphy recorded night time sleep duration and patient reported sleep quality relative to the placebo [128,129]. In another study, melatonin (3 mg/day, 4 weeks double blind, *n* = 18) significantly improved subjective quality of sleep, but did not correct objective sleep abnormalities. The motor dysfunction was not affected by the use of melatonin in this trial [129,130]. Similarly, in a larger study using the same dose, in PD patients with comorbid sleep disorders, melatonin (3 mg/day, 6 weeks double blind, *n* = 38) improved patient reported sleep as measured by the Parkinson’s disease sleep scale (PDSS) in the melatonin group but not placebo treated group. There were significant improvements compared to baseline in sleep latency and total sleep efficiency [131]. Differences between groups were not statistically significant. In addition, in those PD patients, melatonin treatment resulted in cognition improvement and depression test scores (MMSE score, five-word test and the Hamilton scale).

#### 4.2.3. Ramelteon

In a case study, two patients with secondary REM sleep behavior disorder (RBD) complications and Parkinson’s disease were treated with ramelteon and showed an improvement in terms of their clinical RBD symptoms and a decrease in the proportion of REM sleep without atonia [132].

#### 4.2.4. Agomelatine and Tasimelteon

To the best of our knowledge, the efficacy of agomelatine and tasimelteon in treatment of comorbid insomnia in patients with neurological pathologies has not been studied.

## 5. Melatonin Receptor Agonists in Comorbid Cardiovascular Diseases

One of the major health issues found in the 55+ years old population is hypertension [133,134]. The prevalence of hypertension is significantly higher among insomnia patients (~44%) as compared with good sleepers (~19%), suggesting a cross-talk between sleep and blood pressure (BP) control [3]. In particular, higher systolic BP and lower day-to-night systolic BP dipping were reported in normotensive insomniacs as compared with in normotensive good sleepers [135]. Furthermore, short sleep duration and insomnia were found to be risk factors for hypertension, as assessed in middle-aged subjects and depressed patients [136]. In the elderly, it was shown that impaired sleep architecture, as expressed by decreased slow-wave sleep, increases the risk of developing hypertension [137]. The blunted nocturnal BP dip and the resulting nocturnal hypertension have severe consequences and are considered major risk factors for cardiovascular events [138]. Accordingly, a recent Dutch population-based cohort study of 20,432 men and women aged 20–65 years revealed that short sleepers with poor sleep quality had a 63% higher risk of cardiovascular disease (CVD) and a 79% higher risk of coronary heart disease compared with normal sleepers with good quality sleep [139].

Nocturnal hypertension and the absence of blood pressure reduction during sleep (non-dipping) are distinct entities that often occur together and are regarded as important predictors of poor cardiovascular prognosis [140]. Apart from its chronobiotic action, melatonin also directly or indirectly influences a large variety of physiological functions including those of the cardiovascular system. Although the mechanisms of melatonin’s action is still under active investigation, there is substantial evidence indicating cardiovascular effects of melatonin in experimental as well as clinical conditions.

### 5.1. Melatonin Receptor Agonists in Comorbid Cardiovascular Diseases: Preclinical Evidence

Atherosclerosis is a chronic vascular disease in which oxidative stress and inflammation are commonly implicated as major causative factors. Melatonin has atheroprotective effects by acting on different pathogenic signaling processes; these result from its direct free radical scavenger activity, its indirect antioxidant properties and its anti-inflammatory actions [141].

Experimentally induced conditions of reduced melatonin production, such as pinealectomy [142] or continuous light exposure [143], were associated with increased blood pressure. The melatonin-deficiency model of continuous light exposure in rats was associated with left ventricular hypertrophy and fibrosis or an altered collagen composition [143]. While pinealectomy increased the infarct size after ischemia-reperfusion of isolated hearts [144] continuous light exposure increased the susceptibility to ischemia-reperfusion arrhythmias (despite enhancing functional recovery) [145].

Several reports have been published on melatonin antihypertensive effects in experimental models of hypertension, such as spontaneously hypertensive rats (SHR) [146,147] and pinealectomized rats [148].

In SHR, melatonin administration for 5 weeks achieved 25% reduction (44 mmHg) of blood pressure, an effect comparable to spironolactone or simvastatine, but lower compared to captopril (35%) in the same experimental setting [149].

### 5.2. Melatonin Receptor Agonists in Comorbid Cardiovascular Diseases: Clinical Evidence

#### 5.2.1. Melatonin

Low levels of urinary 6-sulphatoxy-melatonin, the major melatonin metabolite, predicted incident hypertension within an 8-year follow-up in young healthy women [150]. Hypertensive patients with insufficient physiological nocturnal blood pressure decline (non-dippers) had lower urinary 6-sulphatoxy-melatonin levels and reduced night/day melatonin concentration ratio compared to dippers [151,152]. In patients with coronary artery disease, serum melatonin levels [153] as well as urinary 6-sulphatoxy-melatonin were reduced [154].

*Post hoc* analysis of pooled antihypertensive drug-treated subpopulations from four randomized, double-blind trials of PRM 2 mg (3 weeks double blind *n* = 392, 28 weeks double blind, *n* = 225) and additional 3 open label studies of PRM (1 year, *n* = 1382) in patients aged 55 years and older with primary insomnia who are treated with antihypertensive drugs indicated significant improvements in Quality of sleep behavior following wakening sleep latency and clinical global impression of Improvement (CGI-I) with PRM (Circadin) compared with placebo. No differences were observed between Circadin and placebo groups in vital signs, including daytime blood pressure at baseline and treatment phases. The rate of adverse events normalized per 100 patient-weeks was lower for Circadin (3.66) than for placebo (8.53). The findings demonstrate substantive and sustained efficacy and safety of PRM for sleep in primary insomnia patients treated with antihypertensive drugs. Circadin appears to be safe for insomnia in patients with cardiovascular comorbidity [155].

Nightly melatonin administration (2.5 mg, fast release, nightly for 3 weeks, double blind, *n* = 16) improved sleep in hypertensive patients treated with the β-blockers atenolol or metoprolol [155]. In comparison with a placebo, three weeks of melatonin supplementation significantly increased total sleep time (mostly sleep stage 2) and sleep efficiency and decreased sleep onset latency assessed by polysomnography. The sleep onset latency remained significantly shortened on the night after discontinuation of melatonin administration, suggesting a carryover effect.

Evidence from the last ten years suggests that melatonin may influence the cardiovascular system in humans [156]. Furthermore, exogenous melatonin has induced several hemodynamic effects in healthy men and women [157,158,159].

Administration of melatonin (1 mg, acute, single blind, *n* = 17) significantly reduced blood pressure, the pulsatile index in the internal carotid artery and catecholamine levels in healthy men and young women 90 min after administration [158,159]. Analysis of four randomized controlled trials of prolonged release melatonin, 2 mg, in patients with insomnia aged 55 and over on stable antihypertensive medication indicated no change compared to placebo in the mean daytime systolic and diastolic blood pressure [160].

Several studies of add-on immediate and PRM preparations to antihypertensive therapy reported amelioration in nocturnal hypertension [161]. In these studies, insomnia was not monitored nor reported. It is therefore important to find out whether the beneficial effects of melatonin on nocturnal hypertension are related to the sleep promoting effects, the circadian clock effects or both. Such preparations would effectively treat insomnia in patients who have cardiovascular comorbidity.

A recent meta-analysis of 7 randomized controlled trials including 344 patients indicated that melatonin supplementation had no significant effect on nocturnal systolic or diastolic blood pressure [162]. However, when subgroup analyses were formed according to formulations employed, controlled-release melatonin (2–3 mg, 4–6 weeks, *n* = 106) was found to significantly and consistently reduce both systolic (by 6.1 mm) and diastolic (by 3.5 mmHg) nocturnal blood pressure compared to placebo treatment. This benefit was not found with fast-release melatonin preparations [162]. Notably, one of the studies included in this meta-analysis even reported that in patients well controlled on nifedipine fast-release melatonin (5 mg) increased systolic/diastolic blood pressure by 6.5/4.9 mmHg respectively compared to placebo suggesting some interaction between melatonin and nifedipine treatment. [163] In any case, the effects of nightly ingestion of melatonin do not impinge on blood pressure during the day.

#### 5.2.2. Agomelatine

Data presented in Section 4 from the European and US studies have not demonstrated that agomelatine is associated with adverse cardiac effects (e.g., ECG or blood pressure changes) [39]. A proposed study comparing the effect of agomelatine and fluoxetine on heart rate variability in patients with major depression has been withdrawn before enrolment (National Taiwan University Hospital, Taipei City, Taiwan) (ClinicalTrials.gov identifier CT00451490]. US National Institutes of Health, ClinicalTrials.gov. (http:// www.clinicaltrials.gov).

#### 5.2.3. Ramelteon

Hiromasa reported that starting early treatment with ramelteon can be beneficial for new cardiovascular disease (CVD) patients [164].

#### 5.2.4. Tasimelteon

To the best of our knowledge, there have not been published that report on the efficacy of tasimelteon in treatment of comorbid insomnia in patients with cardiovascular pathologies.

## 6. Melatonin Receptor Agonists in Comorbid Metabolic Diseases

Diabetes mellitus is a chronic age-related disease affecting an increasing number of patients worldwide and is currently reaching epidemic proportions [165]. Several studies have suggested a direct association between diabetes and sleep disturbances [166,167,168]. Primary sleep disorders have been suggested to promote development of the metabolic syndrome that is strongly associated with increased type-2 diabetes and cardiovascular risk [169].

In several recent studies, a single nucleotide polymorphism of the human melatonin receptor 1B has been described as being causally linked to increased risk of developing type-2 diabetes [170,171,172]. The data suggest that endogenous as well as exogenous melatonin may play a role in improving diabetic control. Abnormalities of the nocturnal melatonin profile have also been described in diabetic patients, mainly in those suffering from diabetic neuropathy [173]. Post mortem studies have indicated an association between diabetes mellitus and decreased melatonin secretion [174]. Melatonin deficiency deprives the brain of an important regulator of sleep and time cue to the internal circadian clock [175] and may thus exacerbate sleep problems in diabetic patients [30].

### 6.1. Melatonin Receptor Agonists in Comorbid Metabolic Diseases: Preclinical Evidence

There is a growing body of evidence suggesting a link between disturbances in melatonin production and impaired insulin, glucose, lipid metabolism, and antioxidant capacity [176,177].

Furthermore, melatonin has been found to influence insulin secretion both *in vivo* and *in vitro* [178]. In models of obesity and the metabolic syndrome, the administration of melatonin not only prevented weight gain, but also reduced insulin and leptin resistance and reduced the susceptibility of the myocardium to ischemia-reperfusion injury [179].

Several studies support that melatonin can prevent hyperadiposity in animal models of obesity. Melatonin treatment of obese old rats ameliorated abdominal obesity, hyperinsulinemia, hypercholesterolemia, hyperglycemia, hyperbetalipoproteinemia and glycosuria [180]. Melatonin can attenuate oxidative stress, lessen liver damage, and improve liver histology in rats with high fat diet-induced non-alcoholic fatty liver disease, when given concurrently with the diet [181]. Melatonin has been shown to decrease the abnormal hyperglycemia seen after a glucose load in 10% fructose-treated rats and also counteracted the changes in plasma low-density lipoprotein-C (LDL-C), triglyceride and cholesterol [182]. These results indicate that melatonin improves metabolic syndrome induced by high fat and high fructose intake in rats.

### 6.2. Melatonin Receptor Agonists in Comorbid Metabolic Diseases: Clinical Evidence

#### 6.2.1. Melatonin

The efficacy and safety of PRM (Circadin 2 mg, 3 weeks cross over, *n* = 36) in the treatment of glucose, lipid metabolism, and sleep was studied in type 2 diabetic patients with insomnia [30]. In an extension period of five months, Circadin was given nightly to all patients in an open-label design. Sleep efficiency, wake time after sleep onset, and number of awakenings assessed by actigraphy improved significantly with Circadin as compared with placebo. No significant changes in serum glucose, fructosamine, insulin, *C*-peptide, antioxidant levels or blood chemistry were observed after 3 weeks of Circadin treatment. Long-term Circadin administration has a beneficial effect on HbA1c, suggesting improved glycemic control [30].

It has been found that the simultaneous application of melatonin with lisinopril or amlodipine (open label study) have a normalizing effect on metabolic parameters affected in 100 elderly patients with arterial hypertension [183]. In another open label study, melatonin administered for two months significantly improved lipid profile (decrease in LDL-C) and lowered blood pressure in 30 metabolic syndrome patients who did not respond to 3-month lifestyle modification in comparison with baseline, particularly in those with arterial hypertension [184].

A recent review on melatonin use in atherosclerosis and dyslipidemia [185] was inconclusive as to whether melatonin can normalize the blood lipid profile at this point [185].

#### 6.2.2. Ramelteon, Agomelatine and Tasimelteon

To the best of our knowledge, there have not been published studies that report efficacy of ramelteon, agomelatine and tasimelteon in treatment of comorbid insomnia in patients suffering from metabolic diseases.

Hence, prolonged-release melatonin may be beneficial and safe for insomnia in diabetic patients. It has still to be determined if metabolic effects beyond sleep are affected in metabolic disorders, and to what extent such effects are specific to melatonin deficiency in certain subpopulations.

## 7. Conclusions

The potential of melatonin as a hypnotic and chronobiotic agent makes the use of melatonin receptor agonists good candidates for induction of physiological sleep in insomnia and circadian rhythm sleep disorders. In addition to their effects in primary sleep disorders, melatonin and its recently introduced agonists are potentially efficacious and safe drugs in the treatment of comorbid insomnia with add-on positive effects in a variety of neurological, psychiatric, cardiovascular and metabolic disorders. The therapeutic value of melatonin receptor agonists in comorbid insomnia deserves further studies.
